# Molecular docking analysis of Aza compounds with the heme-binding protein from *Tannerella Forsythia*

**DOI:** 10.6026/97320630019053

**Published:** 2023-01-31

**Authors:** Janani Sathiamurthy, Gayathri Rengasamy, Surya Sankaran, Kavitha Sankaran, Vishnu Priya Veeraraghavan, Rajalakshmanan Eswaramoorthy

**Affiliations:** 1 Department of Biochemistry, Saveetha Dental College and Hospitals, Saveetha Institute of Medical and Technical Sciences (SIMATS), Saveetha University, Chennai-600077; 2 Department of Biomaterials (Green lab), Saveetha Dental College and Hospital, Saveetha Institute of Medical and Technical Sciences (SIMATS), Saveetha University, Chennai-600077

**Keywords:** Molecular docking, drug discovery, anti-microbial agents, *Tannerella Forsythia*, Aza compounds

## Abstract

The high biological activity and interesting optical properties of the aza compounds is known. Therefore, it is of interest to document the molecular docking analysis data of Aza compounds with the heme-binding protein from an anaerobic, Gram-negative bacterium *Tannerella Forsythia*. Hence, we report the optimal binding features of Aza compounds with the heme-binding protein from *Tannerella Forsythia* for further consideration in drug discovery against the pathogen.

## Background:

*Tannerella Forsythia* is an anaerobic, Gram-negative bacterium. It is the major human bacterial pathogen responsible for periodontitis. These pathogens acquire heme from host hemoproteins using the HmuY hemophore for nutrition and growth. Overgrowth of *T. forsythia* occurs in the subgingival biofilms of periodontally healthy, overweight and obese individuals that might put them at risk for initiation and progression of periodontitis. The periodontal pathogen, *T. forsythia*, was in greater proportions in gingival sulcus in periodontally healthy or gingivitis subjects who were obese, potentially increasing their risk of developing periodontitis. Particularly, younger females who are obese were at greater risk for periodontitis [1]. Therefore, it is of interest to document the computer aided molecular docking analysis data [2,3,4, 5,6] of Aza compounds with the heme-binding protein from *Tannerella Forsythia* for drug discovery.

## Material and Methods:

## Protein preparation:

**Figure 1:**
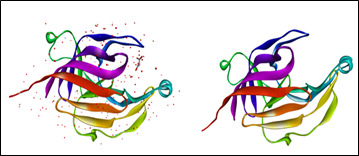
3D structure of Heme binding protein of *Tannerella forsythia* (PDB ID: 6EU8)

The 3D crystal structure of the Heme binding protein (PDB ID: 6EU8) was downloaded from the protein data bank (Figure 1). As per standard protocol, protein preparation was done using the software Biovia Discovery Studio and Mgl tools 1.5.7. Water molecules and cofactors were chosen for elimination. The previously connected ligands were removed, and the protein was produced by adding polar hydrogens and Kollmans charges with Auto Prep.

## Ligand preparations:

**Figure 2:**
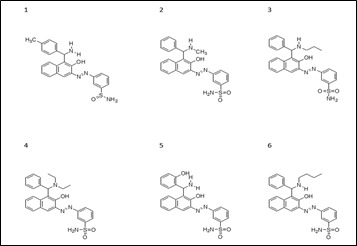
2D Structures of the Aza Compounds (1-6).

The 2D structures of the literature derived Aza compounds are drawn using the ChemDraw 16.0 software (Figure 2). During the optimization method, the software Chem3D was employed and all parameters were selected in order to achieve a stable structure with the least amount of energy. The structural optimization approach was used to estimate the global lowest energy of the title chemical. Each molecule's 3D coordinates (PDB) were determined using optimized structure.

## Auto dock Vina analysis:

**Figure 3:**
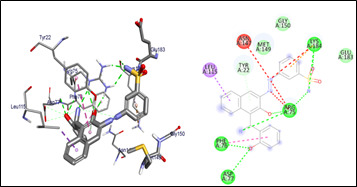
Molecular docking analysis of compound 5 against the target Heme binding protein of *Tannerella forsythia*.

The graphical user interface Auto Dock vina was used for Ligand-Protein docking interactions (Figure 3). Auto Dock Tools (ADT), a free visual user interface (GUI) for the AutoDock Vina software, was used for the molecular docking research. The grid box was built with dimensions 18.1989, 17.3630, 15.5978 pointing in the x, y, and z axes. The central grid box for 6EU8 was 16.6732, 25.4771, 39.5751 A. For each ligand, nine alternative conformations were created and ranked based on their binding energies utilizing Auto Dock Vina algorithms.

## Drug likeness and toxicity predictions:

Pharmacokinetic properties (ADME), drug-likeness, toxicity profiles are examined using SwissADME, and ProTox-II online servers. The SwissADME, a web tool from Swiss Institute of Bioinformatics (SIB) is used to convert the two-dimensional structures into their simplified molecular input line entry system (SMILES). The physicochemical properties (molar refractivity, topological polar surface area, number of hydrogen bond donors/ acceptors); pharmacokinetics properties (GI absorption, BBB permeation, P-gp substrate, cytochrome-P enzyme inhibition, skin permeation (log Kp)) which are critical parameters for prediction of the absorption and distribution of drugs within the body, and drug likeness (Lipinski's rule of five) were predicted using SwissADME. The toxicological endpoints (hepatotoxicity, carcinogenicity, immunotoxicity and mutagenicity) and the level of toxicity (LD50, mg/Kg) are determined using the ProTox-II server.

## Statistical analysis:

One way ANOVA was used for statistical analysis. The clinically proven drugs are used as a control and the results are compared. The significance of the results was found to be p< 0.05

## Results:

## Molecular docking interaction of aza compounds with the Heme binding protein of *Tannerella Forsythia*:

All the compounds (1-6) are run against the target heme bindingprotein of *Tannerella Forsythia* generating docking score range between -5.2 to 8 (Table 1). The compounds show hydrogen molecules interaction similar to clinically proven drug amoxicillin (-6.5). Clinically proven drugs show interaction within the binding site of protein such as ASN, ARG, TYR for amoxicillin and VAL for moxifloxacin. All the compounds show similar binding affinity as the lead molecules that are within the binding site.

## ADME and Lipinski's rule of five:

The compounds show log Kp values between -5.01 to -9.94 cm/s (Table 2). Comparing amoxicillin and moxifloxacin compound 5 shows similar log Kp value. All the compounds show low gastro intestinal absorption so it needs a carrier molecule. Compounds show no blood brain barrier permeability. All the compounds (1-6) obey Lipinski rule of 5 similar to control groups (Table 3).

## Toxicity profiling:

The compounds show class 6 toxicity (Table 4). All the compounds (1-6) show a similar LD50 value (6600 mg/kg). Compound 6 is inactive in hepatotoxicity, carcinogenicity, immunotoxicity, mutagenicity and cytotoxicity. Compound 5 doesn't inhibit the cyt-P450 which is similar to the clinically proven drug hence it can act as a lead molecule.

## Discussion:

Overweight or obese individuals have an overgrowth of *T. forsythia* compared to normal weight individuals, thus subjecting overweight and obese individuals to a higher risk of developing periodontal disease. Complementary strategies involving computational and wet lab experimental approaches help to identify factors that govern interactions of *T. forsythia* with the host as well as other community bacteria [7]. Enrichment in identifying active molecules for the target of interest when compared with random selection is available [8]. It has been widely recognized that drug ADMET properties should be considered as early as possible to reduce failures in the clinical phase of drug discovery [9]. Molecular weight (MW), molecular refractivity (MR), count of specific atom types and polar surface area (PSA) are compiled in physicochemical properties. The PSA is calculated using the fragmental technique called topological polar surface area (TPSA), considering sulfur and phosphorus as polar atoms [10]. Docking scores which are less than -6.5 are the compounds of interest. Compared to the clinically proven drug the selected ligands have better interaction. The selected compounds show more than 2H-bonds within the binding site indicating the stronger interactions and stable complex formation. All the Selected compounds are following Lipinski rule of 5. All the ligands show low Gastro intestinal absorption. They show similar absorption profile like Amoxicillin. All the ligands are skin permeable and there is no Blood Brain Barrier permeation. All compounds show large LD50 value.

## Conclusion:

We report the optimal binding features of Aza compounds with the heme-binding protein from *Tannerella Forsythia* for further consideration.

## Figures and Tables

**Table 1:** Molecular docking interaction of the aza compounds (1-6) against Heme binding protein of Tannerella forsythia (PDB ID: 6EU8).

Ligands	Docking scores/Affinity (kcal/mol)	H-bond	Amino Acid Residual interactions	
			Hydrophobic/Pi-Cation	Van dar Waals
1		LYS-184, ARG-75	TYR-22, LEU-115, GLU-183, PHE-76, ILE-144	GLY-150, ASP-77, LYS-141, MET-149
	-7.9			
2	-7.9	LYS-184, ARG-75, ASN-147	TYR-22, LEU-115	GLY-150, GLU-183, MET-149
3	-7	PHE-118, TYR-22,	ARG-75, LYS-184, MET-149	GLY-117, GLU-23, LYS-21, THR-20, ASN-119, MET-120, ASN-147
4	-8	LYS-184, ARG-75, ASN-147,	TYR-22, PHE-118, LEU-115, ASP-77	GLU-183, GLY-150, PHE-76, MET-114,
5	-7.9	LYS-184, ARG-75, PHE-76, ASP-77,	TYR-22, ASN-147, LEU-115	GLU-183, GLY-150, MET-149,
6	-7.7	LYS-184, ASN-147, ARG-75	ASP-77, LEU-115, PHE-76, TYR-22	MET-114, HIS-74, GLY-150
Amoxicillin	-6.5	ASN-147, ARG-75, TYR-22		LYS-141, GLU-146
Moxifloxacin	-5.9	VAL-145,	LEU-115, ARG-75, ASP-77, ASN-147	TYR-22, HIS-74, ILE-144, GLU-146,
Sulfanilamide	-5.2	ASN-147, ARG-75, TYR-22, ASP-77	PHE-76, LYS-141	
Sulfamethoxazole	-6.2	TYR-22, ASN-147, ARG-75, ASP-77,	LYS-141, PHE-76, LEU-115, PHE-118	

**Table 2:** ADME values of selected aza compounds (1-6)

Compound	log Kp (cm/s)	GI absorption	BBB permeant	Pgp substrate	CYP1A2 inhibitor	CYP2C19 inhibitor	CYP2C9 inhibitor	CYP2D6 inhibitor	CYP3A4 inhibitor
1	-5.42	Low	No	Yes	No	Yes	No	No	No
2	-5.64	Low	No	No	No	Yes	Yes	No	No
3	-5.17	Low	No	No	No	Yes	Yes	Yes	No
4	-5.43	Low	No	Yes	No	Yes	Yes	No	No
5	-6.26	Low	No	No	No	No	Yes	No	No
6	-5.01	Low	No	No	No	Yes	Yes	Yes	No
Amoxicillin	-9.94	Low	No	No	No	No	No	No	No
Moxifloxacin	-8.32	High	No	Yes	No	No	No	Yes	No
Sulfanilamide	-7.79	High	No	No	No	No	No	No	No
Sulfamethoxazole	-7.21	High	No	No	No	No	No	No	No

**Table 3:** Lipinski and Veber rules of selected aza compounds (1-6)

Compound	MW	iLogP	HBD (nOHNH)	HBA (nON)	nrotb	MR	TPSA	Lipinski #violations	Bio availability score
Lipinski*	≤500	≤5	≤5	≤10	≤10	-	-		
Veber**	-	-	-	-	-	-	≤ 140		
1	430.52	3	3	6	5	124.02	133.27	0	0.55
2	446.52	2.95	3	7	6	124.65	125.52	0	0.55
3	474.57	3.6	3	7	8	134.26	125.52	0	0.55
4	488.6	3.35	2	7	8	139.16	116.73	0	0.55
5	448.49	2.82	4	8	5	121.77	159.74	0	0.55
6	488.6	3.35	3	7	9	139.07	125.52	0	0.55
Amoxicillin	365.4	1.46	4	6	5	94.59	158.26	0	0.55
Moxifloxacin	401.43	2.78	2	6	4	114.05	83.8	0	0.55
Sulfanilamide	172.2	0.61	2	3	1	41.84	94.56	0	0.55
Sulfamethoxazole	253.28	1.03	2	4	3	62.99	106.6	0	0.55

**Table 4:** Toxicity profile of selected aza compounds (1-6)

			Toxicity				
Compound	^a^LD_50_ (mg/kg)	Class	HEPATOTOXICITY	CARCINOGENICITY	IMMUNOTOXICITY	MUTAGENICITY	CYTOTOXICITY
1	6600	6	Inactive	Active	Inactive	Inactive	Inactive
2	6600	6	Inactive	Active	Inactive	Inactive	Inactive
3	6600	6	Inactive	Active	Inactive	Inactive	Inactive
4	6600	6	Inactive	Active	Inactive	Inactive	Inactive
5	6600	6	Inactive	Active	Inactive	Inactive	Inactive
6	6600	6	Inactive	Inactive	Inactive	Inactive	Inactive
Amoxicillin	15000	6	Inactive	Inactive	Inactive	Inactive	Inactive
Moxifloxacin	2000	4	Inactive	Inactive	Inactive	Active	Inactive
Sulfanilamide	3000	5	Inactive	Active	Inactive	Inactive	Inactive
Sulfamethoxazole	2300	5	Active	Active	Inactive	Inactive	Inactive
^a^LD_50_: lethal dose parameter
